# Molecular targeted therapy causes hepatic encephalopathy in patients after Transjugular intrahepatic portosystemic shunt (TIPS): A case report and literature review

**DOI:** 10.1016/j.jimed.2021.12.006

**Published:** 2022-02-26

**Authors:** Chen Zhou, Yang Chen, Jiacheng Liu, Qin Shi, Bin Xiong

**Affiliations:** a Department of Radiology, Union Hospital, Tongji Medical College, Huazhong University of Science and Technology, Wuhan, 430022, Hubei, China; b Hubei Province Key Laboratory of Molecular Imaging, Wuhan, 430022, Hubei, China

**Keywords:** Transjugular intrahepatic portosystemic shunt (TIPS), Hepatic encephalopathy, Molecular targeted therapy, Case report

## Abstract

We report two cases of hepatic encephalopathy caused by molecular targeted drugs after the Transjugular intrahepatic portosystemic shunt (TIPS) procedure in our center. The liver toxicities and anti-angiogenic effects induced by targeted drugs may generate an imbalance in ammonia metabolism, elevating blood ammonia levels. TIPS diverts partial blood supply from the liver, aggravates liver impairment, and shunts ammonia-rich blood from the intestine into the systemic circulation. These may be the mechanisms leading to hepatic encephalopathy caused by molecular targeted drugs following TIPS. When clinicians choose molecular targeted therapy as the second or third targeted therapy for patients who have undergone TIPS, the consequence of drug-induced hepatic encephalopathy should also be considered.

## 1 Introduction

Transjugular intrahepatic portosystemic shunt (TIPS) is an effective method for treating cirrhotic portal hypertension, especially upper gastrointestinal bleeding and refractory ascites caused by the rupture of esophageal and gastric varices. However, hepatic encephalopathy may occur after TIPS.^1^ The incidence of hepatic encephalopathy or its aggravation following TIPS has been reported to be 5%–35%.^2^ Hepatic encephalopathy following TIPS is caused by a variety of factors, including a high-protein diet and constipation. However, no studies have reported on hepatic encephalopathy caused by molecular targeted therapy following TIPS. This study focused on two cases of hepatic encephalopathy that were reported in our center in patients who underwent TIPS, caused by molecular targeted drugs. Additionally, by reviewing the literature, this study aimed to discuss the treatment and corresponding mechanisms of hepatic encephalopathy related to this report. The patient has consented to the publication of this manuscript and any identifying images or data.

## 2 Case presentation

**Fig. 1:**
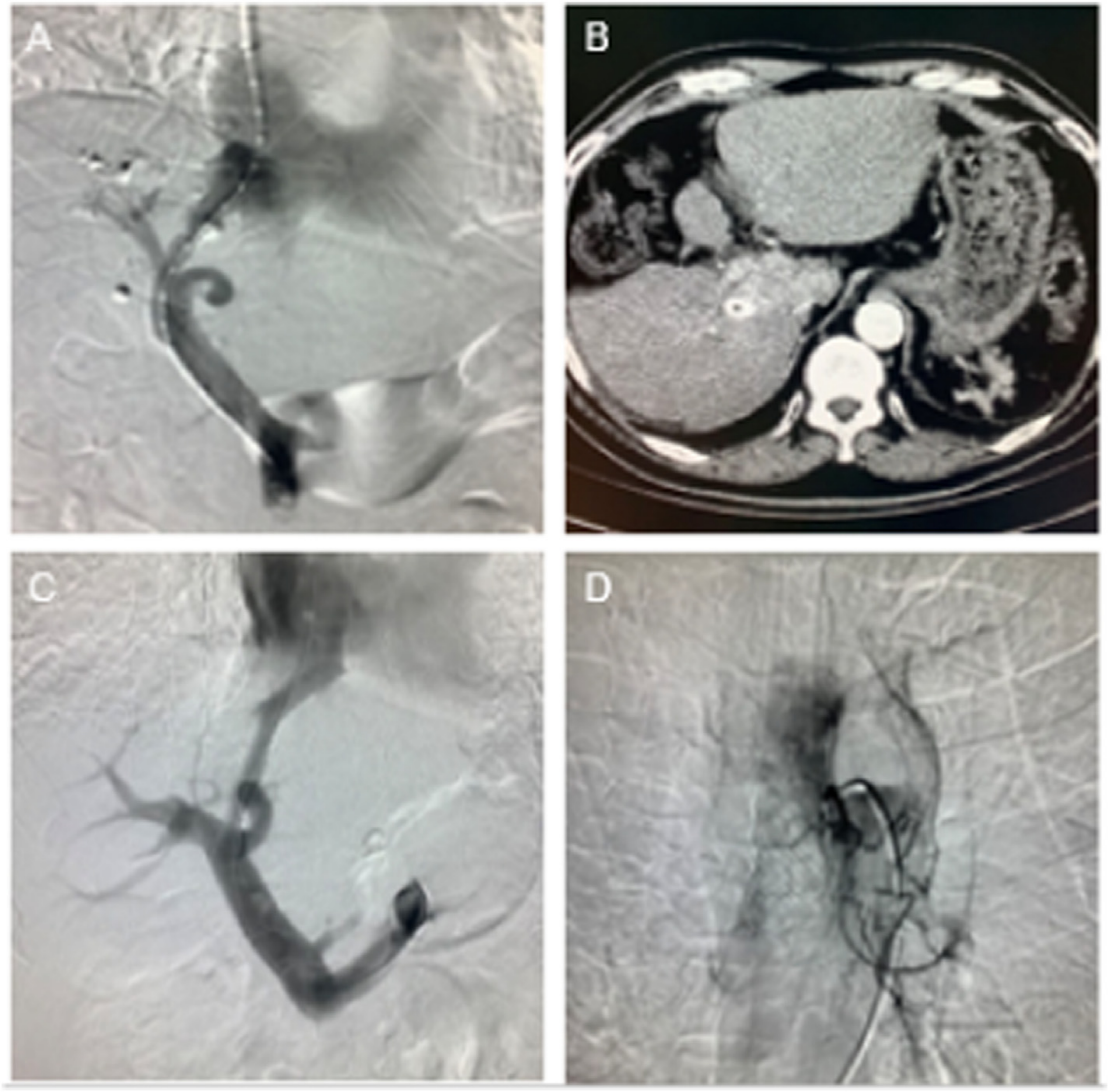
Picture A and B: Digital subtraction angiography (DSA) and postoperative computed tomography (CT) images of the patient with liver cancer combined with portal hypertension. Picture C and D: The TIPS and esophageal arterial chemoembolization DSA images of the patient with esophageal cancer and portal hypertension.

Our first case is a 60-year-old male patient with liver cancer and portal hypertension. He had been treated with multiple transarterial chemoembolization since 2013, combined with targeted therapy using sorafenib at a dosage of 0.2g bid. The status of liver cancer was evaluated as partial remission. The patient underwent TIPS in December 2018 due to gastrointestinal bleeding (Fig. 1A and B). Owing to disease progression, sorafenib was switched to regorafenib at a dosage of 160 mg qd, and the patient was regularly followed-up every 3 months. The second case was a 73-year-old male patient with esophageal cancer and portal hypertension. He underwent TIPS for liver cirrhosis and gastrointestinal bleeding in 2019 and recovered well after the procedure. Because of esophageal cancer with dysphagia, he underwent stent placement in August 2020, followed by arterial chemoembolization combined with oral apatinib at a dosage of 500mg qd to treat esophageal cancer, and the patient was regularly followed-up every 3 months. (Fig. 1C and D).

Both patients had no symptoms of drowsiness before taking the targeted drugs, and their plasma ammonia levels were within the normal range. Moreover, their liver function was classified as Child–Pugh class A (Table 1). Symptoms of hepatic encephalopathy, such as cognitive dysfunction, elevated blood ammonia level, and coma, occurred within a week of taking the targeted drug, and their liver function worsened (Table 1). Both patients were diagnosed with grade 4 hepatic encephalopathy after excluding other possible causes of brain dysfunction according to the West Haven criteria.^1^ The targeted drugs were stopped immediately, and intravenous infusion of branched-chain amino acids, ornithine aspartate, was administered to lower blood ammonia levels, refreshing the brain, and lactulose was administered orally to induce catharsis. The patient's consciousness became clear, and their blood ammonia levels and liver function returned to normal (Table 1). These two patients were subsequently treated with interventional therapy and symptomatic treatment, and the targeted drugs were not rechallenged. Therefore, the patient no longer exhibited symptoms of hepatic encephalopathy.

**Table 1:** Changes in blood ammonia and liver functions of two patients.

Patient 1	Pre-operation	Post-operation	Before taking drugs	After taking drugs	Drug withdrawal
blood ammonia (umol/L)	35	36	9	148	30
ALT (U/L)	51	101	14	119	15
AST (U/L)	62	87	26	48	28
Child-Pugh score	5	5	5	6	5
Patient 2					
blood ammonia (umol/L)	48	36	18	111	36
ALT (U/L)	55	256	14	59	25
AST (U/L)	115	378	39	68	46
Child-Pugh score	5	5	5	7	5

## 3 Discussion

The increasing use of TIPS and the prolonged survival time of patients after surgery may lead to cancer following TIPS.^3^^,^^4^ The two patients in our center had portal hypertension and tumors. Targeted antitumor treatments were administered following TIPS, and symptoms of hepatic encephalopathy appeared that improved after discontinuation of the drug. Therefore, it was evident that the symptoms of hepatic encephalopathy, such as lethargy and fatigue, were caused by the intake of targeted drugs. Molecular targeted therapy is a revolutionary treatment approach that halts cancer growth, progression, and metastasis by interfering with specific molecules.^5^ Many molecular targeted therapies approved by the United States Food and Drug Administration have achieved significant clinical efficacy in treating liver, lung, esophageal, colorectal, and other cancers. Owing to their anti-angiogenic effect, molecular targeted drugs can also inhibit the growth of the cerebral vascular endothelium, causing increased permeability of the blood–brain barrier.^6^ Consequently, high levels of ammonia cross the blood–brain barrier and disrupt the glutamate and glutamine pathways in the astrocytes of the brain, leading to neurological disorders.^7^ This may be one of the mechanisms leading to hepatic encephalopathy.

The liver is the main site of ammonia metabolism. The liver toxicities induced by targeted drugs may generate an imbalance in ammonia metabolism, elevating the blood ammonia level.^1^ TIPS diverts partial blood supply from the liver, aggravates liver impairment, and shunts ammonia-rich blood from the intestine into the systemic circulation.^8^ The use of targeted drugs reduces the blood flow to the liver and worsens liver function.^9^ Poor liver function and hepatotoxicity caused by these drugs have also been suggested as possible reasons for the occurrence of hepatic encephalopathy. Events that lead to increased levels of ammonia in the blood or brain have been implicated to worsen hepatic encephalopathy, whereas reducing blood ammonia levels improves hepatic encephalopathy.^10^^,^^11^ Therefore, special attention should be paid to the development of hepatic encephalopathy when targeted drugs are used.

In conclusion, an increase in serum ammonia levels remains important to our understanding of hepatic encephalopathy, and therapies remain directed toward lowering ammonia levels in patients with signs of hepatic encephalopathy. When clinicians choose molecular targeted therapy as the second or third targeted therapy for patients who have undergone TIPS, we should have the diagnosis and treatment consciousness to detect mild hepatic encephalopathy, and drug-induced hepatic encephalopathy should be considered to avoid iatrogenic triggers.

## Financial support

This work was funded by 10.13039/501100001809the National Natural Science Foundation of China (81873917).

## Declaration of competing interest

The authors declare that they have no known competing financial interests or personal relationships that could have appeared to influence the work reported in this paper.
